# An integrin binding motif in TLN-1/talin plays a minor role in motility and ovulation

**DOI:** 10.17912/micropub.biology.000726

**Published:** 2023-01-04

**Authors:** Fereshteh Sadeghian, Ibrahim Ibrahim, Lokesh Ravichandran, Grace Henderson, Anisha Acharya, Lianzijun Wang, Myeongwoo Lee, Erin J. Cram

**Affiliations:** 1 Northeastern University, Department of Bioengineering, Boston, MA, USA; 2 Department of Biology, Baylor University, Waco, TX, USA; 3 Northeastern University, Department of Biology, Boston, MA, USA

## Abstract

TLN-1/talin is a conserved focal adhesion protein that forms part of the linkage between the cytoplasmic tail of integrin and the actin cytoskeleton. In *C. elegans* , TLN-1 is expressed strongly in striated muscle and the gonadal sheath cells. Here, we report that a CRISPR-generated TLN-1 allele TLN-1(W387A), predicted to affect binding of talin to integrins, results in mild phenotypes, including motility defects and ovulation defects. The arrangement of the actin cytoskeleton in the body wall muscles, spermatheca, and sheath appears identical in wild type and TLN-1(W387A) animals. This analysis suggests that W387 in TLN-1 does not have a major effect on the binding of talin to integrin *in vivo* .

**Figure 1. TLN-1(W387A) results in modest motility and oocyte transit defects:**
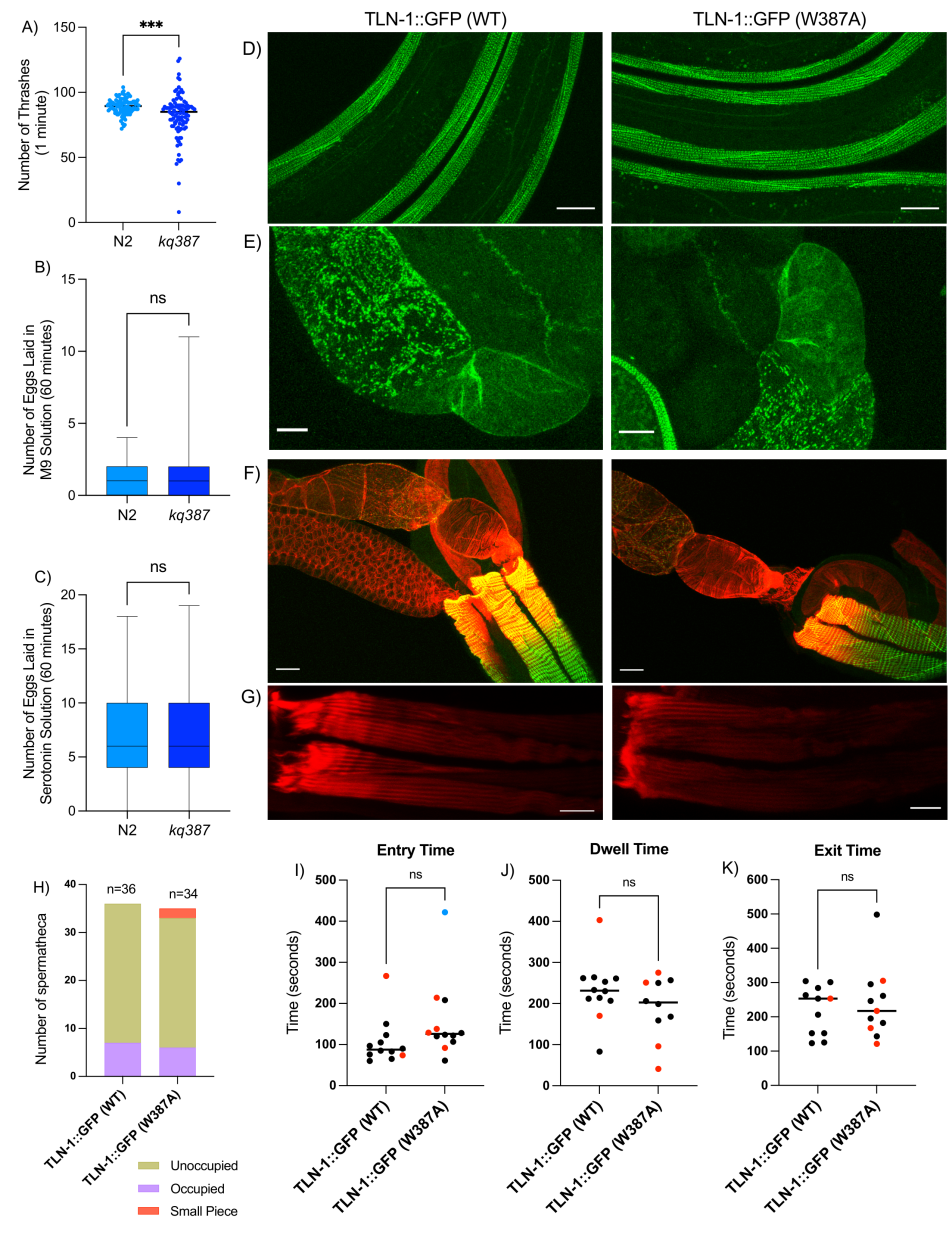
A) The *tln-1(kq387)* strain BU387 thrashed less on average compared to N2 (Student’s t-test; p<0.0002). B) No significant difference in egg laying in M9 solution was observed between BU387 *tln-1(kq387)* and N2 (Student’s t-test p = 0.7620). C) No significant difference in stimulated egg laying in 3 mg/mL serotonin creatine sulfate monohydrate solution was observed between BU387 tln-1(kq387) and N2 (Student’s t p-value of 0.9828). D-E) Confocal microscopy of the AH3437 TLN-1::GFP(WT) and BU388 TLN-1::GFP(W387A) animals indicating talin expression in body wall muscles (D), spermatheca, and sheath (E), scale bar= 20 mm. F-G) Phalloidin staining indicates actin alignment in spermatheca, sheath, germline, and body wall muscle scale bar= 20 mm. H) Spermathecal occupancy ‘trapping screen’ scoring of TLN-1::GFP(WT) and TLN-1::GFP(W387A) indicates the presence or absence of an embryo in the spermatheca. I-K) Assessment of timing of oocyte transit through the spermatheca (oocyte entry, dwell, and exit time) of of TLN-1::GFP(WT) and TLN-1::GFP(W387A) animals (blue: fertilized oocyte, red: oocyte fragment 'small piece').

## Description

TLN-1/Talin is a conserved focal adhesion protein that activates the integrins to bind extracellular matrix (ECM) and links the cytoplasmic tail of integrin to the actin cytoskeleton (Moulder et al., 1996), forming a critical attachment point between ECM and the cytoskeleton. Talin engages the NPxY (asp-pro-x-tyr) domain of β-integrin via its FERM (F3) domain. Structural analysis of mouse talin 1 showed that altering tryptophan W359 to alanine (W387 in TLN-1) in the talin head domain resulted in the loss of interaction between talin and the NPXY motif in the β-integrin cytoplasmic tail (Zhang et al., 2020). In *C. elegans* , TLN-1 is expressed in skeletal muscle and somatic gonad and is predicted to engage the β-integrin PAT-3, which contains the conserved NPXY motif in its cytoplasmic tail (García-Alvarez et al., 2003). Downregulation of TLN-1 expression by RNAi leads to motility defects including paralysis and muscle degradation, and gonad morphogenesis and ovulation defects (Cram et al., 2003; Lecroisey et al., 2007). Although the *tln-1* null phenotype is not known, a deletion allele *n4099* (which also removes *mir-50* ) is inviable (Miska et al., 2007).

In order to study the role of this conserved tryptophan (W387) *in vivo* , the W to A mutation was induced using the CRISPR-Cas9 into N2 animals. The CRISPR edit was confirmed by sequencing. To our surprise, the homozygous TLN-1(W387A) mutant, *tln-1(kq387)* , is superficially wild type. Thrashing assays can reveal subtle body wall muscle contractility defects (Lesanpezeshki et al., 2021). We measured the number of body thrashes per minute in a drop of M9 buffer. In the motility assays, the number of thrashes per minute in *kq387* and N2 were significantly different (Figure 1A, *p* <0.0002), suggesting TLN-1(W387A) may play a role in body wall muscle function. We next explored a possible effect of TLN-1(W387A) on the egg laying muscles, however, no significant difference in egg laying was observed between N2 nor *tln-1(kq387)* in M9 buffer (Figure 1B), or when stimulated by adding 3 mg/ml serotonin in M9 (Figure 1C).

We also introduced the W387 edit into AH3437, an endogenously labeled TLN-1::GFP strain, and confirmed the edit by sequencing. Confocal microscopy of the TLN-1(WT)::GFP and TLN-1(W387A)::GFP animals revealed similar TLN-1 expression patterns in the body wall muscles, spermatheca, and sheath (Figure 1D and E). In body wall muscle, TLN-1::GFP (WT) is localized to dense bodies, M-lines and the adhesion plaques of muscle cell boundaries. These data suggest that residue W387 is not needed for TLN-1 localization in *C. elegans* . Because talin plays an important role in attachment to actin, we explored whether TLN-1 W387 is required for assembly or maintenance of the actin cytoskeleton in the spermatheca. F-actin staining with Texas Red phalloidin indicated similar actin alignment in spermatheca, sheath, and germline (Figure 1F) and body wall muscle (Figure 1G) of the TLN-1(WT)::GFP and TLN-1(W387A)::GFP animals.

The spermatheca is a contractile tube in *C. elegans* that is composed of 24 smooth muscle-like cells. Mature oocytes become fertilized in the spermatheca and pass through the spermathecal-uterine (sp-ut) valve and into the uterus. We scored embryo transits through the spermatheca, noting occupied or unoccupied spermathecae or occupation by a small piece of an embryo which indicates severing of the oocyte upon entry. TLN-1(W387A)::GFP animals exhibited significantly more severed ‘small piece’ oocytes than TLN-1(WT)::GFP animals (Figure 1H). Transit parameters including the entry time, the amount of time the embryo remained completely enclosed within the spermatheca ‘dwell time’, and the exit time were not significantly different (Figure 1 I-K).

These results suggest that TLN-1(W387A) produces measurable phenotypes in motility and in ovulation, however, the phenotypes are mild in comparison to *tln-1(RNAi)* which results in paralysis, sterility, and progressive disruption of the actin cytoskeleton (Cram et al., 2003). It is possible more severe motility defects could be revealed through whole animal locomotion assays on plates. In multiple systems, the talin recruits vinculin to integrin-based adhesions (Klapholz & Brown, 2017). In future studies, it would be interesting to explore whether TLN-1 also localizes DEB-1/vinculin to dense body structures, and, if so, whether W387A plays a role in this interaction. However, our current data suggests TLN-1(W387A) likely retains significant ability to bind to integrin and connect integrin to the actin cytoskeleton. The expression of TLN-1::GFP is strongest in the body wall muscle and gonadal sheath cells, with lower levels in the spermatheca. This suggests the ‘small piece’ defect may be primarily due to lack of coordination between the sheath and the spermatheca during oocyte entry, with the spermathecal entry neck closing prematurely around the oocyte and severing it.

## Methods

***C. elegans* strains and culture**

*C. elegans* were grown on NGM plates (0.107 M NaCl, 0.25% wt/vol Peptone, 1.7% wt/vol BD BactoAgar, 2.5 mM KPO4, 0.5% Nystatin, 0.1 mM CaCl _2_ , 0.1 mM MgSO4, 0.5% wt/vol cholesterol) and fed with *E. coli OP50* at 23°C. *C. elegans* AH3437 (TLN-1::GFP) (Walser et al., 2017) and N2 strains were obtained from the *C. elegans* Genetics Center. The mutation was induced in N2 wild type worms using the CRISPR/Cas9 system for DNA sequence editing. Briefly, the mixture sgRNA (TLN1W387), co-sgRNA (ZQDP10A), HDR ssDNA template (TLN1W387A), tracrRNA (#1072532), and Cas9 riboprotein (cat. #1081058) were microinjected into the distal arm of the hermaphrodite gonad. F1 animals with Dpy phenotypes were isolated and genotyped for the presence of desired W387A mutations by using the single-worm PCR method. The F2 progeny from the F1 mutants were further screened to isolate homozygous animals. Homozygous animals were outcrossed to N2 males multiple times to remove non-specific targeting and generate strain BU387 *tln-1(bu387)* . To visualize the TLN-1 distribution, AH3437 GFP:: *tln-1* was also microinjected with the injection mix mentioned above with a mixture containing sgRNA (TLN1W387) to generate strain BU388.

PCR was used to verify the genomic modification. Briefly, single animals were lysed in 6 μl worm lysis buffer (10 mM Tris HCl pH 8.3, 50 mM KCl, 2.5 mM MgCl2, 0.45% Tween 20, 0.45% Triton X100) and PK (Proteinase K) and frozen at -80℃. After protease digestion at 60℃ for 1 hr and denaturation at 95℃ for 15 min, the lysate was used for PCR with GoTaq green master mix using forward primer (talin_W387A_F2: gcgggagaagagacgcagact) and reverse primer (talin_W387A_R: CCTCGCCAACTCCTGCGATGT) to amplify the *tln-1* locus. The edits were verified by sequencing.

**Thrashing and egg laying assays**

Behavioral assays were performed on the mutant and N2 animals. A motility ‘thrashing’ assay was conducted to observe the effects of the *tln-1(kq387)* mutation on muscle and neurons. N2 and BU387 *tln-1(kq387)* worms (n=100) were immersed in 50 μl M9 buffer solution. After one minute of acclimation, body bends were counted for sixty seconds. For the egg-laying assay, N2 or *kq387* young hermaphrodites were placed in microtiter plate wells containing 100 µl M9 or 100 µl of 3 mg/ml serotonin solution. The number of eggs laid in each sample was determined at 15 minutes (for acclimation) and 75 minutes (final egg counting).

**Spermathecal occupancy and transit assays**

To prepare age matched young adult animals for the spermathecal occupancy and transit assays, gravid hermaphrodites were lysed in an alkaline hypochlorite solution to release eggs, which were then placed onto seeded NGM plates and grown at 23°C for 52 hours. Animals were mounted on 5% agarose in 0.1 M sodium azide and observed immediately with DIC microscopy to score spermathecal occupancy rates. Imaging was performed on a Nikon Eclipse 80i microscope with a 60× oil-immersion lens using SPOT Advanced software (Version 5.3.5) and a charge-coupled device camera. To assess transit of embryos through the spermatheca, young adult hermaphrodites were immobilized with a 1:3 ratio of M9 buffer and 0.05 μm Polybead microspheres on a 5% agarose pad for video imaging. Frames were captured at a rate of 1 Hz. Image stacks were reassembled and analyzed using ImageJ software. Entry time is the time from beginning of entry until the oocyte is completely enclosed within the spermatheca. Dwell time is the total amount of time the embryo remained enclosed by the spermatheca. Exit time is the time from opening of the sp-ut valve until the embryo is expelled into the uterus.

**Fluorescent confocal imaging**

Texas Red-X phalloidin staining of F-actin staining was adapted from Ono et al. (2007). Briefly, animals were dissected using a 25-gauge hypodermic needle in phosphate-buffered saline (PBS), and dissected gonads were fixed in 1.85% formaldehyde in PBS for 25 min at room temperature. After fixation, gonads were washed twice with PBS, permeabilized for 15 min in PBST (PBS + 0.1% Triton X-100), and then incubated with 0.4 U/ml Texas Red-X–phalloidin in PBS (Invitrogen, Carlsbad, CA) overnight at 4°C. Labeled samples were washed twice with PBS and mounted on 2% agarose pads for observation. For imaging of GFP::TLN-1, animals were dissected and mounted as described above. Confocal microscopy of animals was performed on a Zeiss LSM 710 confocal using Zen software and a Plan-Apochromat 63x/1.40 oil objective lens using a 488-nm laser (Wirshing & Cram, 2017).

**Image analysis and Statistics**

ImageJ software (version 2.3.0/1.53f) was used to analyze the ovulation movies (Kelley et al., 2020). GraphPad Prism (version 9.2.0) was used for the statistical analysis. The Student’s t-test and the Fisher’s Exact test were performed for comparing movement, egg laying, transit timepoints and proportions of occupied spermatheca as indicated.

## Reagents

**Table:** 

Oligo Name	Sequence	Usage
TLN1W387ASEQF	GCCATAGTTCTTGAAATATTTTGCC	CRISPR
TLN1W387ASEQR	ATCTGTCCGTGAGCAACCAG	CRISPR
TLN1W387WTF	ACTGGAGCAAGTTCGCCGATGG	CRISPR
TLN1W387AF	TAGAACAGGTGAGGCGTGCC	CRISPR
talin_W387A_F2	GCGGGAGAAGAGACGCAGACT	Sequencing
talin_W387A_R	CCTCGCCAACTCCTGCGATGT	Sequencing
GFP_671F	TGGAAGCGTTCAACTAGCAG	Sequencing
talin_100R	CATCGCGAATTTTTCCCTAA	Sequencing
TLN1W387	CACTGGAGCAAGTTCGCCGA	CRISPR crRNA
Repair Template TLN1W387A	GAGAATTCCAAGCAAATCCTCAAAGAATGGCCACTAGAACAGGTGAGGCGTGCCGTTCCCTCTGCGAAATGCTTCTCCCTCGACTTTGG	CRISPR
Strain	Genotype	
N2	Wild type	
AH3437	*GFP::tln-1*	
BU387	*tln-1(kq387)*	
BU388	*GFP::tln-1(kq387)*	
